# RIG-I inhibits pancreatic β cell proliferation through competitive binding of activated Src

**DOI:** 10.1038/srep28914

**Published:** 2016-06-28

**Authors:** Yi Pan, GuangMing Li, HengGao Zhong, MeiJuan Chen, TingTing Chen, LiLi Gao, HuiWen Wu, Jun Guo

**Affiliations:** 1 Department of Biochemistry and Molecular Biology, Nanjing Medical University, Nanjing, PR China; 2 Department of Anesthesiology, Huaian First People’s Hospital, Nanjing Medical University, Huaian, Jiangsu, PR China; 3 Medical Center for Digestive Diseases, Second Affiliated Hospital, Nanjing Medical University, Nanjing, Jiangsu, PR China; 4 The Pre-clinical Medicine College, Nanjing University of Chinese Medicine, Nanjing, PR China; 5 Laboratory Center for Basic Medical Sciences, Nanjing Medical University, Nanjing, PR China

## Abstract

Nutrition is a necessary condition for cell proliferation, including pancreatic β cells; however, over-nutrition, and the resulting obesity and glucolipotoxicity, is a risk factor for the development of Type 2 diabetes mellitus (DM), and causes inhibition of pancreatic β-cells proliferation and their loss of compensation for insulin resistance. Here, we showed that Retinoic acid (RA)-inducible gene I (RIG-I) responds to nutrient signals and induces loss of β cell mass through G1 cell cycle arrest. Risk factors for type 2 diabetes (e.g., glucolipotoxicity, TNF-α and LPS) activate Src in pancreatic β cells. Elevated RIG-I modulated the interaction of activated Src and STAT3 by competitive binding to STAT3. Elevated RIG-I downregulated the transcription of *SKP2*, and increased the stability and abundance of P27 protein in a STAT3-dependent manner, which was associated with inhibition of β cell growth elicited by Src. These results supported a role for RIG-I in β cell mass loss under conditions of metabolic surplus and suggested that RIG-I-induced blocking of Src/STAT3 signalling might be involved in G1 phase cycle arrest through the Skp2/P27 pathway in pancreatic β cells.

Type 2 diabetes mellitus (T2DM) is increasing at an alarming rate and has become a global challenge. This increase has resulted from the improvement in living conditions and increased intake of carbohydrate, fat and meat in the daily diet, especially for Asian populations. Over-nutrition, and resulting obesity, both trigger chronic inflammation in metabolic tissues (termed meta-inflammation) and contribute directly or indirectly to β-cell dysfunction, both quantitatively (i.e., the relative decrease in β-cell mass) and qualitatively (impaired β-cell insulin secretion), which result in compromised insulin secretion and T2DM^1^. Promoting β cell proliferation and their mass compensation associated with the demand for insulin would aid the prevention of type 2 diabetes development efficiently^2^.

Pattern recognition receptors (PRRs) are involved in anti-proliferation activities in pancreatic β cells during nutrition metabolism and meta-inflammation, indicating a pivotal role of PRRs in metabolic regulation and T2DM^3^. Retinoic acid (RA)-inducible gene I (RIG-I) is a typical intracellular PRR. Upon viral dsRNA challenge, RIG-I is activated and regulates the expressions of various inflammatory cytokines to initiate innate and adaptive immunity to protect the organism. In addition, RIG-I is involved in cell proliferation, differentiation and apoptosis. There is an Src-interacting motif, PxxP, in the N-terminal caspase activation and recruitment domain of RIG-I. The PxxP motif combines with the Src SH3 domain to prevent Src from associating with its downstream substrate^4^.

Src, a non-receptor tyrosine kinase, is activated by many factors, such as high glucose, palmitate, leptin, TNF-α and IFN-γ^5^,^6^,^7^,^8^,^9^. Activation of Src might result in the promotion of survival and proliferation pathways, and even induce malignant tumours (v-src)^10^. Many cell-cycle regulatory proteins involved in cell proliferation are induced by Src, such as cyclin A, E, D1, A/CDK2 complex, P27 and P53^11^,^12^,^13^,^14^. Active Src induces phosphorylation of P27, and then downregulates its protein level^15^. Protooncogene Src was suggested to counteract the reduction of Skp2 protein effectively, which involves the negative regulation of P27 and plays a proto-oncogenic role *in vitro* and *in vivo*^16^,^17^. In addition, the transcription factor STAT3 can be activated by Src to regulate Skp2 expression positively^18^. However, the effects of tyrosine kinases on cell-cycle regulatory proteins in pancreatic β cells remain unclear.

In the context of obesity or glucolipotoxicity, PRRs in the immune system are activated and are involved in metabolic modulation^19^,^20^. Glucolipotoxicity and meta-inflammation play critical roles in the development of type 2 diabetes and result in Src activation^6^, together with elevated RIG-I. According to Kim *et al*., inflammatory responses linked with innate immunity might be affected by obesity^21^, and RIG-I has been reported to be elevated during the innate immune response^22^; therefore, our study aimed to explore the potential association between the two signal molecules.

## Materials and Methods

### Reagents

Dulbecco’s Modified Eagle’s medium (DMEM), Roswell Park Memorial Institute 1640 (RPMI1640) and foetal bovine serum (FBS) were purchased from Gibco (Carlsbad, CA, USA). Lipofectamine 2000 and Trizol were bought from Invitrogen (Carlsbad, CA, USA). The Reverse Transcription Kit and the SYBR Green PCR Master Mix were obtained from Takara (Otsu, Shiga, Japan). 3-[4,5-dimethylthiazol-2-yl]-2,5 diphenyl tetrazolium bromide (MTT), Type V collagenase, Histopaque-1077 and palmitate were bought from Sigma Aldrich (St. Louis, MO, USA). TNFα was obtained from Perprotech (Rocky Hill, NJ, USA). The Cell-Light™EdU DNA Cell Proliferation Kit was obtained from RiboBio (Guangzhou, China). The STAT3 dependent reporter construct pGMSTAT3-Lu was from Genomeditech (Shanghai, China). RIPA lysis buffer, the nuclear and cytoplasmic protein extraction kit, the BCA kit and ortho- Nitrophenyl-β-galactoside (ONPG) were obtained from Beyotime Inc (Shanghai, China). The antibodies against RIG-I, Cyclin D1, Cyclin E and Cdk2 were purchased from cell signaling technology (Boston, MA, USA). The antibodies against β actin and insulin were obtained from BOSTER (Wuhan, China). The antibodies against P21, P27, Src, phospho-Src (Tyr418), STAT3 and phospho-STAT3 were obtained from EnoGene (Enogene Biotech Co. Ltd., China). The SKP2 antibody was purchased from Bioss (Beijing, China). The antibodies against Fluorescein-Conjugated AffiniPure Goat Anti-Rabbit IgG and Rhodamine (TRITC)–conjugated AffiniPure Goat Anti-Mouse IgG were bought from ZSGB-BIO (Beijing, China). The Recombinant Human IGF-1 antibody was from PEPROTECH (Rocky Hill, NJ, USA).

### Cell culture and transfection

The pancreatic β cell line MIN6 was cultured in DMEM (4.5 g/l glucose) supplemented with 15% (v/v) FBS, 121 μmol/l 2-mercaptoethanol, 100 U/ml penicillin and 100 μg/ml streptomycin^23^. Transient transfections of plasmid constructs and siRNAs were performed using Lipofectamine 2000, according to the manufacturer’s instructions.

### Islet isolation, culture and *in vitro* transfection

Ten-week-old (20–25 g) male imprinting control region (ICR) mice and db/db mice were obtained from the Model Animal Research Center of Nanjing University. Animals were housed individually in standard polycarbonate cages with sawdust bedding. Water and food were available ad libitum. Principles of laboratory animal care were followed and all procedures were conducted according to the guidelines established by the National Institutes of Health. Every effort was made to minimize suffering. This study was approved by the Research Animal Care Committee of Nanjing Medical University (Nanjing, China). Islet isolation and culture techniques were as previously described^24^. The isolated islets were transferred to and cultured in serum free transfection medium (Ca^2+^-containing Krebs-Ringer-HEPES medium) at 2-d post-isolation, *in vitro* transfection was conducted as previously described^25^.

### Protein isolation and western blotting

The BCA kit was used to determine the protein concentrations. Isolated mouse islets and whole cell lysates of pancreatic β cell lines were prepared for western blotting using various primary antibodies, as indicated. Secondary antibodies against rabbit or mouse IgG were used to detect protein signals^26^.

### Co-immunoprecipitation

1–2 μg of antibody was added to the same amount (400 μg) of cell lysates overnight at 4 °C. Protein A/G-agarose spheres (Santa Cruz Biotechnology, Dallas, TX, USA) were added and the samples stored at 4 °C for 2 h. The samples were then centrifuged at 14,000 × *g*, 4 °C for 2 min, washed with lysis buffer three times and boiled for 10 min after the addition of 20 μL 5 × SDS Loading Buffer. The denatured samples were kept at −20 °C for western blotting^27^.

### Quantitative real-time reverse transcription polymerase chain reaction (qRT-PCR) analysis

Total RNA was isolated using the Trizol reagent. One microgram of total RNA was converted into first-strand cDNA using a Reverse Transcription Kit. SYBR Green and the 7300 Real-Time PCR system (Applied Biosystems, Carlsbad, CA, USA) were used to carry out the qRT-PCR analysis. PCR cycling conditions included initial denaturation at 95 °C for 30 s; 40 cycles of denaturation at 95 °C for 5 s, primer annealing and extension at 60 °C for 34 s; followed by final extension at 68 °C for 45 s. β-actin gene expression was used as an internal standard to calculate the expression levels. The specific primers used are shown in Table S1.

### Cell viability

MIN6 cells were seeded in 96-well plates at 1 × 10^4^ cells/well for MTT measurement and then subjected to the indicated treatments. Thereafter, 20 μL of 5 mg/mL MTT was added to each well and incubated for 4 h. The formazan crystals were dissolved in dimethyl sulphoxide after the supernatant was removed. A microplate reader was used to measure the absorbance at 490 nm to assess the cell viability^28^.

### Cell proliferation assay by 5-ethynyl-2′-deoxyuridine (EdU) labelling

DNA synthesis was analysed using an EdU Labelling Kit. MIN6 cells were cultured in 6-well plates on coverslips. EdU was added to the culture medium (50 μM) for 2 h after treatment and cell proliferation was determined according to the manufacturer’s instructions.

### Immunofluorescence assay (IFA)

IFA was used to observe visually the changes in localization and levels of phospho-STAT3 or the level of Src. MIN6 cells or isolated mouse islets were subjected to IFA after transfection and pharmaceutical treatment. Fluorescein-labelled antibodies diluted in PBS-BSA [anti-insulin/Cy3 antibody (1:50) and anti-phospho-STAT3/TRITC antibody (1:50) or anti-insulin/Cy3 antibody (1:50) and anti-Src/TRITC antibody (1:50)] were added to MIN6 cells or mouse islets and incubated overnight at 4 °C. Isolated mouse islets in suspension were centrifuged at 2000 × g for 5 min at 4 °C at each step^23^,^26^.

### Flow cytometric analysis

A trypsin-EDTA solution was used to digest MIN6 cells. Trypsinised MIN6 cells were collected by centrifugation at 500 × *g* for 5 min. The MIN6 cell pellets were washed with PBS three times and fixed in cold 75% ethanol at 4 °C overnight. Flow cytometry, preceded by propidium iodide (PI) staining, was used to determine the percentages of cells in the G0/G1, S and G2/M phases.

### Luciferase reporter assay

According to the manufacturer’s instructions, the luciferase reporter construct pGMSTAT3-Lu was transfected transiently into MIN6 cells cultured in 24 well plates, using the Lipofectamine 2000 reagent. The gene encoding β galactosidase, expressed in a plasmid driven by the cytomegalovirus (CMV) promoter (Clontech Laboratories, Palo Alto, CA, USA), was transfected simultaneously as an internal control. Six hours after transfection, the medium was replaced. The cells were treated 24 h after transfection and harvested for luciferase reporter assays, as described previously^29^.

### Data analysis

All data were representative of at least three experiments. Results are expressed as the mean ± SEM. Comparisons were performed using Student’s t-test for two groups or ANOVA for multiple groups. P values < 0.05 were considered statistically significant.

## Results

### Src is activated in MIN6 cells

**Figure 1:**
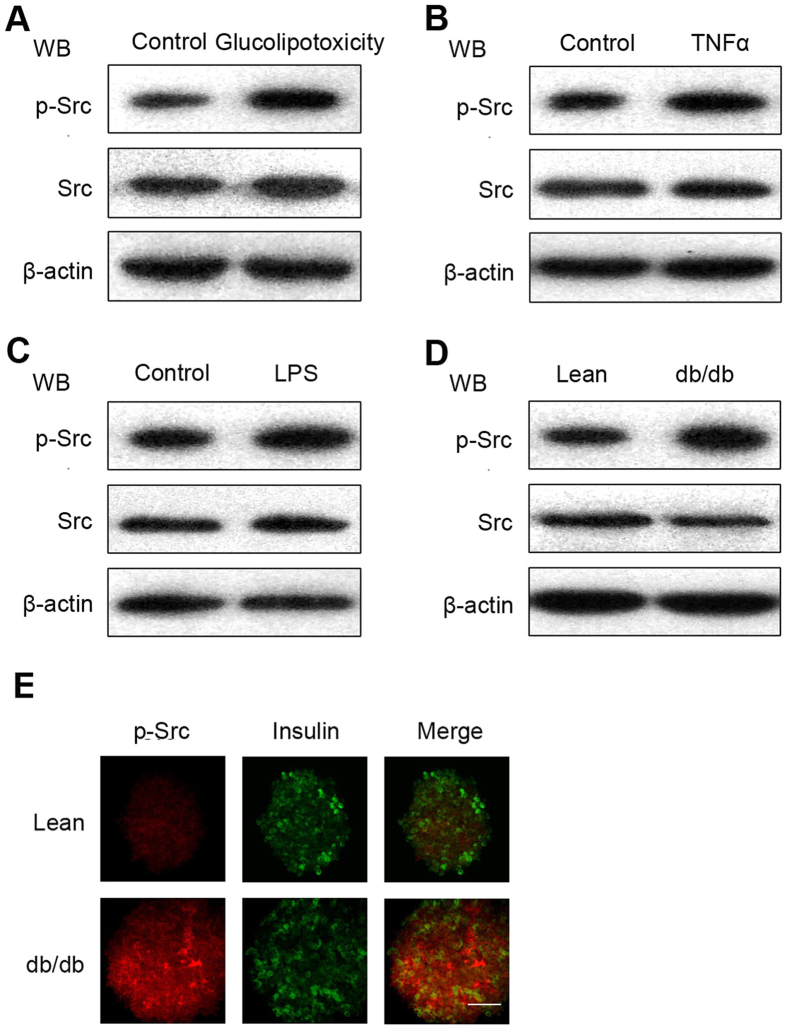
Src is activated in rodent models of type 2 diabetes. Min6 cells were treated with: (**A**) glucolipotoxicity (glucose 16.7 mM and 0.4 mM palmitate) for 24 h; (**B**) TNF-α (80 nM) for 6 h; or (**C**) LPS (10 μg/mL) for 24 h, and cell extracts were collected. (**D**) Isolated islets from ICR or db/db mice. Cell extracts were analysed by western blotting using antibodies against p-Src (Tyr416) and Src. β-actin was detected as an internal control. (**E**) Islets were isolated from ICR or db/db mice. IFA was performed with antibodies directed against p-Src (red) and insulin (green). Insulin with green staining was used to identify β cells (scale bar = 100 μm). Data are means ± SEM of three separate experiments.

To explore the association of Src with β-cells mass and T2DM, especially its risk factors such as Glu-palm, LPS and TNF-α, MIN6 cells were subjected to different stimuli, such as treatment with 0.4 mM palmitate plus 16.7 mM glucose for 24 h, or 80 nM TNF-α for 6 h, or 10 μg/mL LPS for 24 h, according to our previous reports^28^,^30^. The protein levels of Src and p-Src were then assessed by western blotting (Fig. 1A, B,C). The results showed an increase of p-Src in MIN6 cells that were exposed to glucolipotoxicity, or treated with TNF-α or LPS (P < 0.05), while the protein level of Src was stable (P > 0.05). Primary islets were isolated from male db/db mice (originally bred from C57BL/6J mice) and normal male ICR mice (C57BL/6J). The protein level of p-Src was also increased in primary islets from db/db mice compared with the control mice (Fig. 1D, P < 0.05), suggesting that Src is activated *in vivo* in response to glucolipotoxicity. Activated Src in primary islets was also examined using IFA with an anti-p-Src antibody (red fluorescence Fig. 1E). Activated Src was significantly elevated in islets isolated from male db/db mice compared with normal male ICR mice (C57BL/6J). An anti-insulin antibody was employed to distinguish islet β cells from non-β cells in this experiment. The data suggested that Src tyrosine kinases are activated in MIN6 cells in response to risk factors for the development of T2DM.

### Proliferation of pancreatic β cells is inhibited in rodent models of T2DM

**Figure 2:**
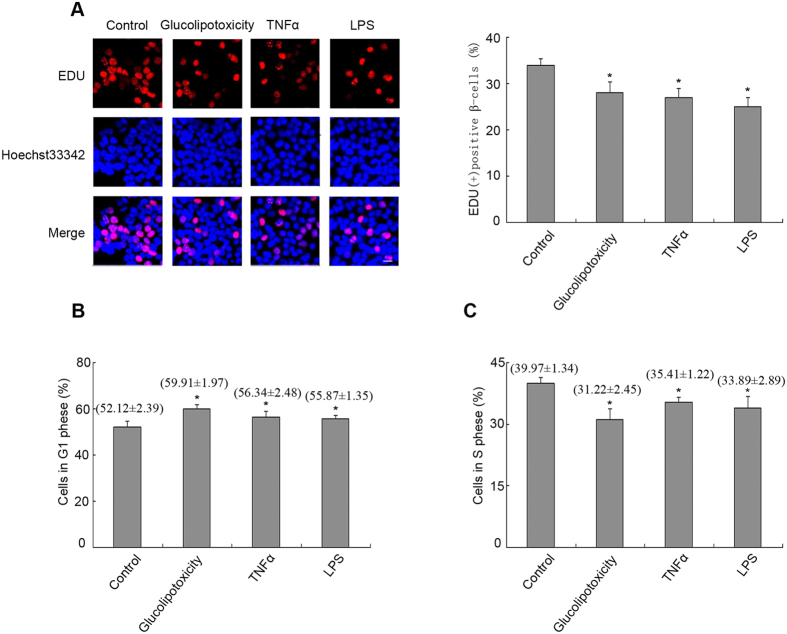
Proliferation of pancreatic β cells is inhibited in rodent models of type 2 diabetes. Min6 cells were stimulated with glucolipotoxicity, TNF-α and LPS for 24 h, as indicated in Fig. 1. (**A**) DNA synthesis was analysed using EdU labelling assays. Representative micrographs of EdU labelling assays in MIN6 cells are shown (scale bar = 120 μm). The percentage of EdU-positive β cells was quantified. Flow cytometric assays were performed to determine the percentages of MIN6 cells at the (**B**) G1 phase and (**C**) S phase. Data are means ± SEM of three separate experiments. *P < 0.05 versus control.

We next investigated whether activated Src is involved in the proliferation of pancreatic β cells in response to the stimuli related to T2DM. After exposure of MIN6 cells to glucolipotoxicity (0.4 mM palmitate plus 16.7 mM glucose), TNF-α (80 nM) or LPS (10 μg/mL) for 24 h^28^,^30^, the percentage of EdU-positive β cells decreased compared with the control (Fig. 2A, P < 0.05). Meanwhile, flow cytometry after PI labelling showed that the percentage of MIN6 cells increased in the G1 phase (Fig. 2B, P < 0.05), but decreased in the S phase (Fig. 2C, P < 0.05) in response to glucolipotoxicity, TNF-α or LPS. These data indicated that proliferation did not increase after the cells were treated with factors related to T2DM. These factors led to cellular senescence via G1 cell cycle arrest in MIN6 cells. Considering the data in Fig. 1, we hypothesized that activated Src did not improve cell viability and proliferation in MIN6 cells in response to the risk factors of T2DM.

### RIG-I is upregulated in rodent models of T2DM

**Figure 3:**
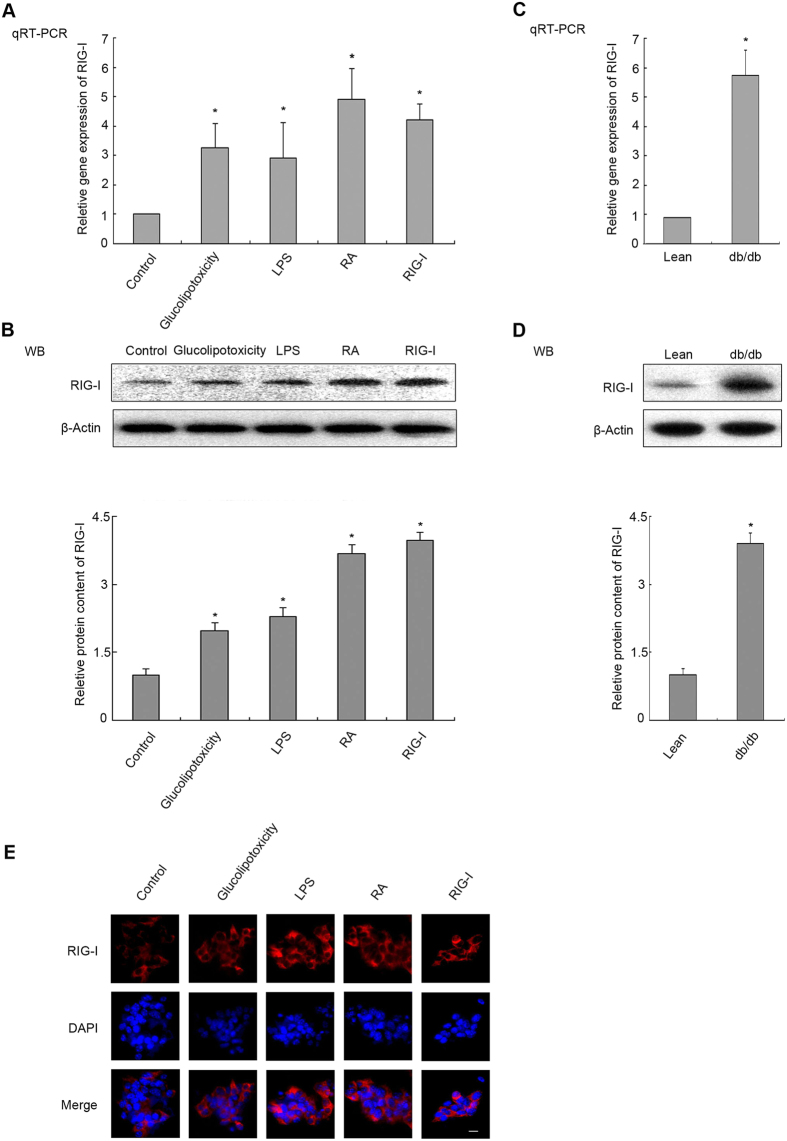
RIG-I is upregulated in MIN6 cells. Min6 cells were stimulated with glucolipotoxicity, LPS or retinoic acid (RA) (10 μM), or transfected with a plasmid encoding *RIG-I* for 24 h, as indicated, and cell extracts were collected. (**A**) QRT-PCR to detect the mRNA levels of RIG-I in Min6 cells. (**B**) Cell extracts were analysed by western blotting using antibodies against RIG-I. Islets were isolated from ICR or db/db mice. (**C**) QRT-PCR analysis and (**D**) western blotting were performed to detect the mRNA or protein levels of RIG-I in primary cells. Quantification of the relative protein content of RIG-I is shown. β-actin was detected as an internal control. (**E**) Min6 cells were treated with glucolipotoxicity, LPS or RA or transfected with a plasmid encoding RIG-I for 24 h, as indicated. IFA was performed with antibodies against RIG-I (red); DAPI was used for nuclear staining (blue) (scale bar = 120 μm). Data are means ± SEM of three separate experiments. *P < 0.05 versus control.

To explore the relationship between the PRR RIG-I and risk factors related to T2DM, cells were exposed to risk factors of T2DM. Additionally, retinoic acid (RA) is a specific agonist of RIG-I. In MIN6 cells exposed to glucolipotoxicity (0.4 mM palmitate plus 16.7 mM glucose), LPS (10 μg/mL) or RA (20 μM) for 24 h, or transfected with plasmid RIG-I, qRT-PCR analysis indicated that the mRNA level of *RIG-I* was obviously increased (Fig. 3A, *P < 0.05). Western blotting showed that glucolipotoxicity, LPS, RA or transfection with RIG-I remarkably increased the RIG-I protein levels (Fig. 3B P < 0.05). The mRNA level and protein level of RIG-I were also increased in primary islets from db/db mice compared with the corresponding controls (Fig. 3C,D; P < 0.05), suggesting that RIG-I increased in response to *in vivo* glucolipotoxicity. Meanwhile, RIG-I was also examined by IFA using an anti-RIG-I antibody (red fluorescence, Fig. 3E). RIG-I was increased in cells treated with glucolipotoxicity, LPS, RA or RIG-I-transfection compared with the control group. These data showed that RIG-I was upregulated in rodent models of T2DM, accompanied by activated Src signals.

### Elevated RIG-I inhibits proliferation in a pancreatic β cell line

**Figure 4:**
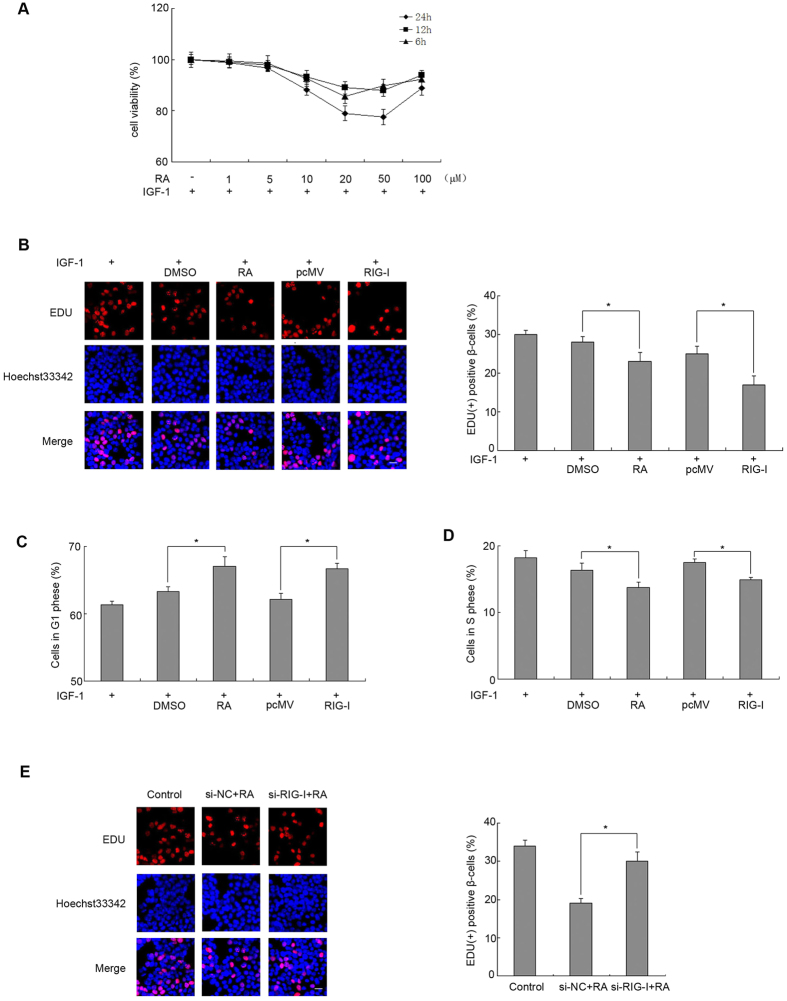
Elevated RIG-I inhibits proliferation in pancreatic β cell line. (**A**) Min6 cells were treated with the indicated concentrations (0, 1, 5, 10, 20, 50 and 100 μM) of retinoic acid (RA) for the indicated times (6 h, 12 h and 24 h) and stimulated with IGF-1 (10 nM). Cell viability was assessed by the MTT assay. 24 hours after transfection of a plasmid encoding *RIG-I*, Min6 cells were treated with IGF-1 and RA as indicated. (**B**) DNA synthesis was analysed using EdU labelling assays. Representative micrographs of EdU labelling assays in MIN6 cells are shown (scale bar = 120 μm). The percentage of EdU-positive β cells was quantified. Flow cytometric assays were performed to calculate the percentages of MIN6 cells at the (**C**) G1 phase and (**D**) S phase. (**E**) After transfection of si-RIG-I for 24 h, Min6 cells were treated with RA. DNA synthesis was analysed using EdU labelling assays. Representative micrographs of EdU labelling assays in MIN6 cells are shown (scale bar = 120 μm). The percentage of EdU-positive β cells was quantified. Data are means ± SEM of three separate experiments. *P < 0.05 versus control.

Insulin-like growth factor I (IGF-1) was reported to induce cell proliferation, and is closely associated with Src activity^31^. To explore the role of RIG-I in Src signalling and cell proliferation in pancreatic β cells, we performed a series of experiments using IGF-1 treatment. RA, a RIG-I specific agonist, was used in the experiment at different concentrations (0, 1, 5, 10, 20, 50 and 100 μM) for different times (6, 12 and 24 h) with IGF-1 (10 nM)^28^ to investigate the role of RIG-I in the pancreatic β cell line. MTT assays indicated that RA at more than 5 μM inhibited the viability of MIN6 cells in a dose-dependent manner (Fig. 4A). We then investigated whether RIG-I could inhibit the proliferation of pancreatic β cells. MIN6 cells were subjected to starvation for 24 h in medium depleted of amino acids and serum, followed by IGF-I treatment in serum-free medium^28^, and then treated with RA (10 μM) or transfected with plasmid RIG-I. The percentage of EdU-positive β cells increased in the cells treated with IGF-1 only, while the percentage of EdU-positive β cells decreased in the cells treated with IGF-1 and RA or in those transfected with plasmid RIG-I (Fig. 4B, P < 0.05). Flow cytometric assays after PI labelling showed that the percentage of MIN6 cells increased in the G1 phase (Fig. 4C, P < 0.05), but decreased in the S phase in response to RA treatment or RIG-I plasmid transfection (Fig. 4D, P < 0.05). To explore whether RIG-I affects the proliferation of β cells, we used a small interfering RNA, si-RIG-I RNA, to transfect MIN6 cells. The percentage of EdU-positive cells increased in cells transfected by si-RIG-I RNA and treated with RA compared with cells transfected with a scrambled siRNA (Fig. 4E, P < 0.05). These results suggested that RIG-I contributes to the inhibition of proliferation elicited by IGF/Src signalling in MIN6 cells.

### Elevated RIG-I inhibits proliferation via stabilization of P27 in pancreatic β cell line

**Figure 5:**
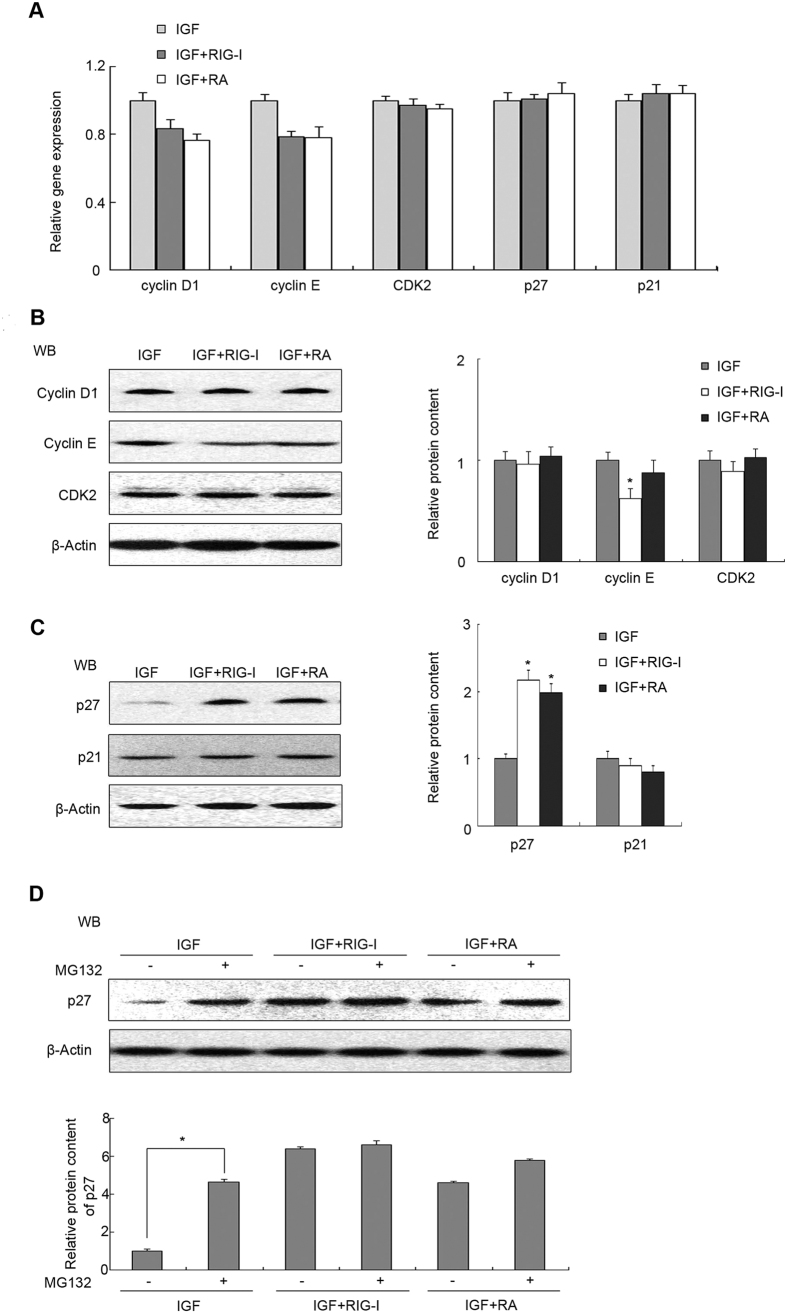
Elevated RIG-I inhibits proliferation via stability of p27 in pancreatic β cell line. 24 h after transfection of a plasmid encoding *RIG-I*, Min6 cells were treated with IGF-1 and RA, as indicated. (**A**) The mRNA levels of the indicated genes, including those encoding positive cell cycle regulators cyclin D1, cyclin E, CDK2, and negative regulators p21 and p27, were determined by qRT-PCR. Cell extracts were analysed by western blotting with antibodies against (**B**) positive and (**C**) negative cell cycle regulators. (**D**) MIN6 cells were pre-treated with or without MG132 (20 μM) for 1 h and subsequently treated as indicated. Cell extracts were analysed by western blotting with antibodies against P27. β-actin was detected as an internal control. Data are means ± SEM of three separate experiments. *P < 0.05 versus control.

Elevated RIG-I inhibits Src-induced proliferation and the phenomenon is associated with the cell cycle. Thus, cyclin D1, cyclin E, CDK2, P27Kip1 and p21 were analysed at the mRNA and protein levels. MIN6 cells were subjected to starvation for 24 h in medium depleted of amino acids and serum, followed by IGF-I treatment in serum-free medium. Cells were transfected with plasmid RIG-I or treated with RA. QRT-PCR analysis indicated that the cyclin D1 and cyclin E mRNAs decreased slightly and CDK2, p27 and p21 mRNA levels remained stable (Fig. 5A, P > 0.05). Meanwhile, western blotting showed that RIG-I transfection induced a decrease in cyclin E protein levels and increased P27 levels (Fig. 5B,C, P < 0.05), whereas other protein levels were unaffected (P > 0.05). To explore the possible involvement of the RIG-I pathway in β-cells upon IGF-induced downregulation of P27 protein levels, MG132, a specific inhibitor of the proteasome, was used a subsequent experiment. MG132 remarkably upregulated the protein levels of P27Kip1 in response to IGF stimuli (Fig. 5D, P < 0.05), but did not affect the change of P27Kip1 when RIG-I was elevated (Fig. 5D, P > 0.05). These data suggested that RIG-I-induced accumulation of P27Kip1 might be attributed to increased stability of P27 and impairment of ubiquitin-dependent proteasome degradation of this protein.

### RIG-I induced downregulation of Skp2, which is involved in the stability of P27 in pancreatic β cells

**Figure 6:**
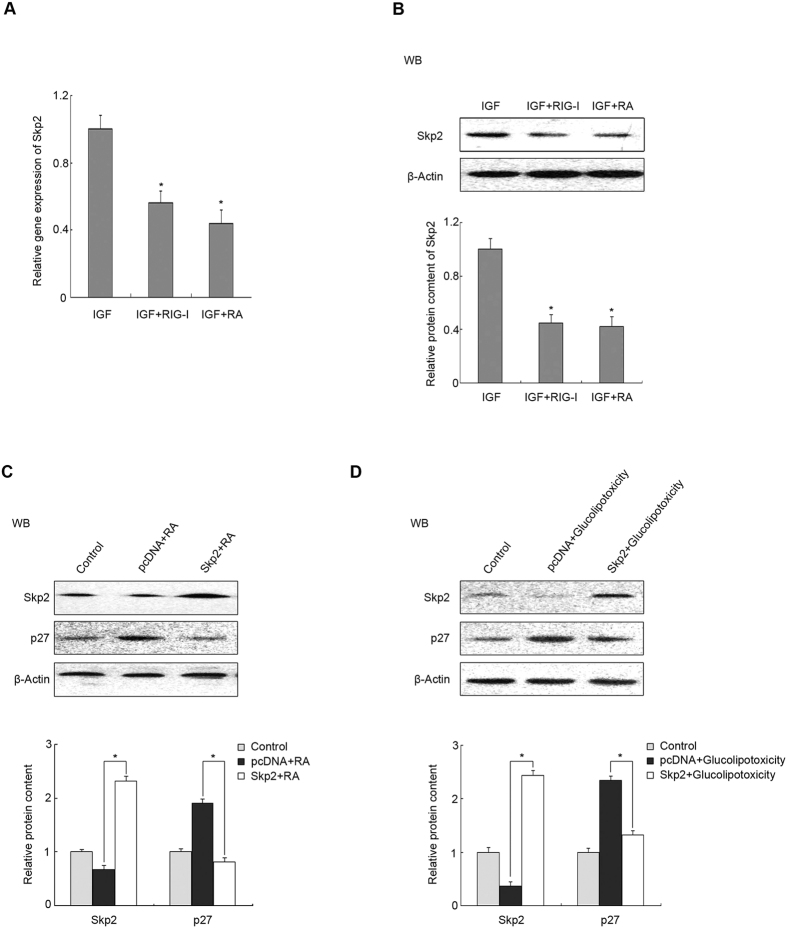
RIG-I-induced downregulation of Skp2 is involved in the stability of P27 in a pancreatic β cell line. 24 h after transfection of plasmid encoding *RIG-I*, Min6 cells were treated with IGF-1 and RA, as indicated in the above experiments. (**A**) The mRNA and (**B**) protein levels of *Skp2* were determined by qRT-PCR and western blotting, respectively. Min6 cells were transfected with a plasmid encoding *Skp2* for 24 h and subsequently treated with (**C**) RA or (**D**) glucolipotoxicity, as indicated. Cell extracts were analysed by western blotting with antibodies against Skp2 and P27. β-actin was detected as an internal control. Data are means ± SEM of three separate experiments. *P < 0.05 versus control.

The periodicity of P27 depends on ubiquitin degradation, and the specificity of ubiquitination is provided by the E3 ligases. Skp2 is an E3 ligase that targets P27. To confirm whether Skp2 is involved in this process, IGF-1, RA and plasmid with RIG-I were used to treat MIN6 cells. QRT-PCR and western blotting analyses showed that the *SKP2* mRNA and protein levels were reduced (Fig. 6A,B) after MIN6 cells were treated with RA or transfected with the RIG-I plasmid, which were significantly different to cells treated with IGF-1 only (P < 0.05). To explore the role of Skp2 in P27 protein stability, MIN6 cells were transfected with a *SKP2* plasmid. *SKP2* overexpression reversed RA-induced upregulation of P27 protein levels (Fig. 6C, P < 0.05), suggesting that RIG-I regulates P27’s function through Skp2-induced protein degradation. Glucolipotoxicity is involved in the increment of RIG-I protein levels and proliferation inhibition of pancreatic β-cells; therefore, to explore the molecular mechanisms underlying role of RIG-I in glucolipotoxicity-induced proliferation inhibition, MIN6 cells were treated using 0.4 mM palmitate plus 16.7 mM glucose. The Skp2 protein level decreased and P27 protein level increased in response to glucolipotoxicity. Skp2 overexpression could reverse the increment in the P27 protein level elicited by glucolipotoxicity (Fig. 6D, P < 0.05). These data indicated that RIG-I induced downregulation of the Skp2 protein level, resulting in increased stability of P27, which might be associated closely with glucolipotoxicity.

### STAT3 increased the expression of Skp2 in a pancreatic β cell line

**Figure 7:**
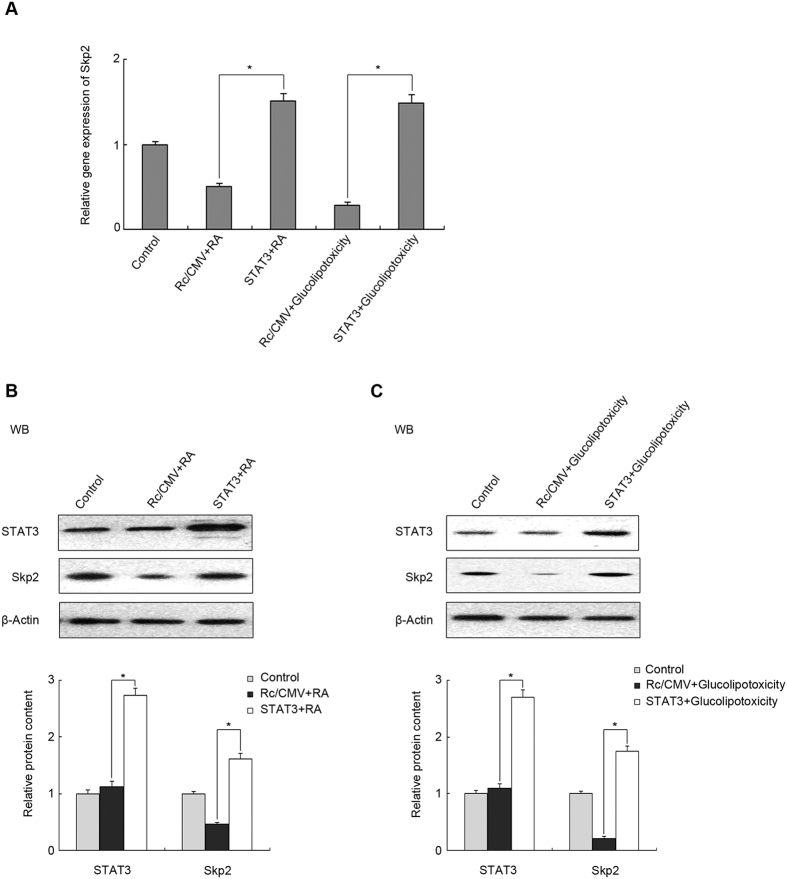
STAT3 increased the expression of Skp2 in pancreatic β cell line. Min6 cells were transfected with a plasmid encoding STAT3 for 24 h and subsequently treated with RA or glucolipotoxicity, as indicated. (**A**) The mRNA level of *Skp2* was determined by qRT-PCR. Min6 cells were transfected with plasmid encoding *Skp2* for 24 h and subsequently treated with (**B**) RA or (**C**) glucolipotoxicity, as indicated. Cell extracts were analysed by western blotting with antibodies against STAT3 and Skp2. β-actin was detected as an internal control. Data are means ± SEM of three separate experiments. *P < 0.05 versus control.

RIG-I affected the expression of *SKP2* mRNA level. Previous literature showed that STAT3 might induce Skp2 expression^32^; therefore, to explore the mechanism underlying altered *SKP2* transcription, we used plasmid STAT3 to transfect MIN6 cells. After transfection, RA or glucolipotoxicity were used to stimulate the cells. QRT-PCR and western blotting analyses showed that STAT3 overexpression could reverse the RA-induced downregulation of *SKP2* mRNA and protein levels (Fig. 7A,B, P < 0.05), suggesting that STAT3 is involved in the regulation of *SKP2* transcription. To explore the molecular mechanisms underlying the role of STAT3 in glucolipotoxicity-induced dysfunction of pancreatic β-cells, MIN6 cells were treated using 0.4 mM palmitate plus 16.7 mM glucose. STAT3 overexpression reversed the reduced mRNA and protein levels of Skp2 in response to glucolipotoxicity (Fig. 7A,C, P < 0.05). These data suggested that STAT3 could be an important regulator controlling *SKP2* gene expression in pancreatic β-cells.

### Transcriptional activity of STAT3 was suppressed by RIG-I accumulation

**Figure 8:**
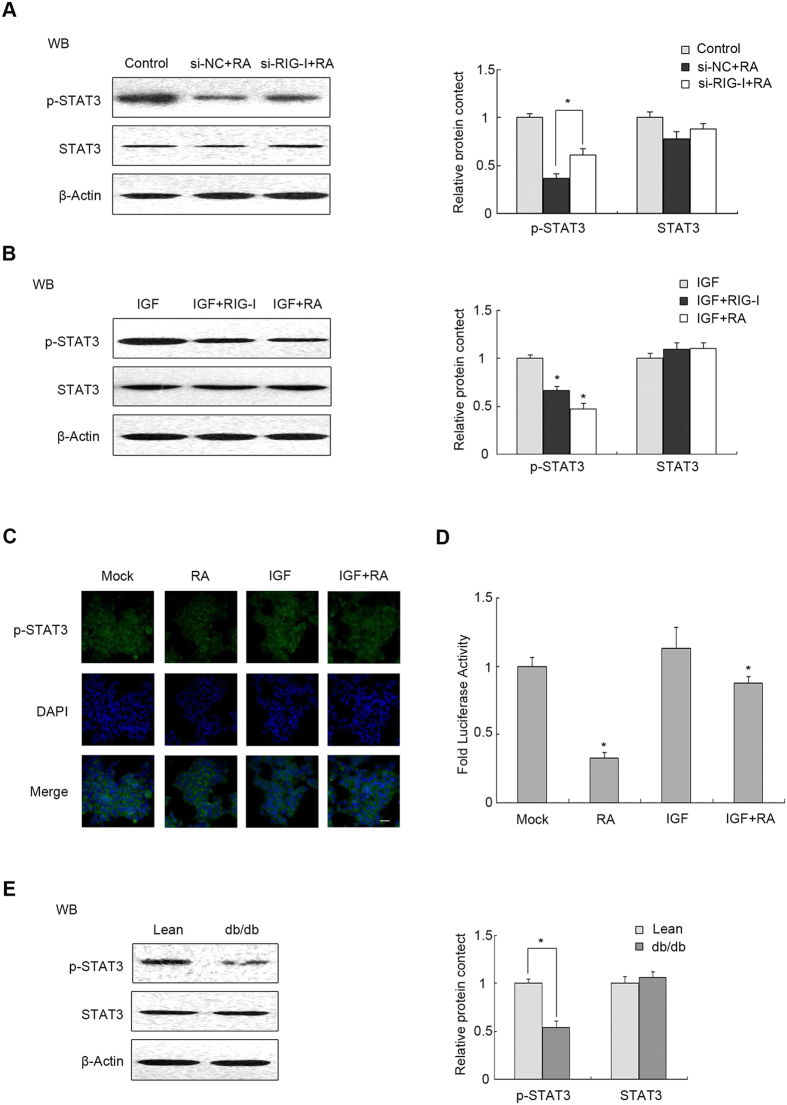
Transcriptional activity of STAT3 was suppressed by RIG-I accumulation. (**A**) Min6 cells were transfected with si-RIG-I for 24 h, and then treated with RA. Protein levels of p-STAT3 and STAT3 were determined by western blotting. (**B**) 24 h after transfection of a plasmid encoding *RIG-I*, Min6 cells were treated with IGF-1 and RA, as indicated. Western blotting was used to detect the protein levels of p-STAT3 and STAT3. (**C**) Min6 cells were treated with IGF-1 or RA, as indicated. IFA was performed with an antibody against p-STAT3 (green); DAPI was used for nuclear staining (blue) (scale bar = 100 μm). (**D**) Luciferase activity of pGMSTAT3-Lu (a STAT3-dependent reporter) was measured and shown as the fold change. (**E**) Islets were isolated from ICR or db/db mice. Protein levels of p-STAT3 and STAT3 were determined by western blotting in primary islet cells. β-actin was detected as an internal control. Data are means ± SEM of three separate experiments. *P < 0.05 versus control.

STAT3 contains an Src homology 2 (SH2) domain that is activated by tyrosine phosphorylation in response to a wide variety of cytokines and growth factors^33^. To explore the relationship between STAT3 and factors that influence Src signals, cells were subjected to different treatments. We used si-RIG-I to transfect MIN6 cells and treated the cells with RA. The protein level of p-STAT3 decreased after the cells were treated with RA only, and si-RIG-I plasmid transfection reversed the decrease (Fig. 8A, P < 0.05). We then used IGF-1 to treat MIN6 cells grown in serum-free DMEM. Plasmid RIG-I and RA were used to treat the cells. The protein level of p-STAT3 was inhibited more in cells treated with IGF-1 and RA or transfected with plasmid RIG-I compared with IGF-1 treatment only (Fig. 8B, P < 0.05). Using IFA with an anti-p-STAT3 antibody (green fluorescence Fig. 8C), p-STAT3 was observed to be increased significantly in the nucleus when treated with IGF-1 compared with the mock group grown in serum-free DMEM; however, it was reduced in cell nucleus when treated with RA. To explore the *STAT3* transcription activity, MIN6 cells were transfected with a STAT3-dependent reporter construct pGMSTAT3-Lu and then treated as in Fig. 8B. Compared with the mock group or IGF-1 treatment, the luciferase activity was reduced significantly in the group subjected to RA (Fig. 8D, P < 0.05). The protein level of p-STAT3 was also decreased in primary islets from db/db mice compared with the corresponding control (Fig. 8E, P < 0.05), suggesting that STAT3 is activated in response to *in vivo* glucolipotoxicity. These data indicated that RIG-I was involved in the transcriptional activity of *STAT3* in a phosphorylation-dependent manner.

### RIG-I interrupts the binding between Src and STAT3

**Figure 9:**
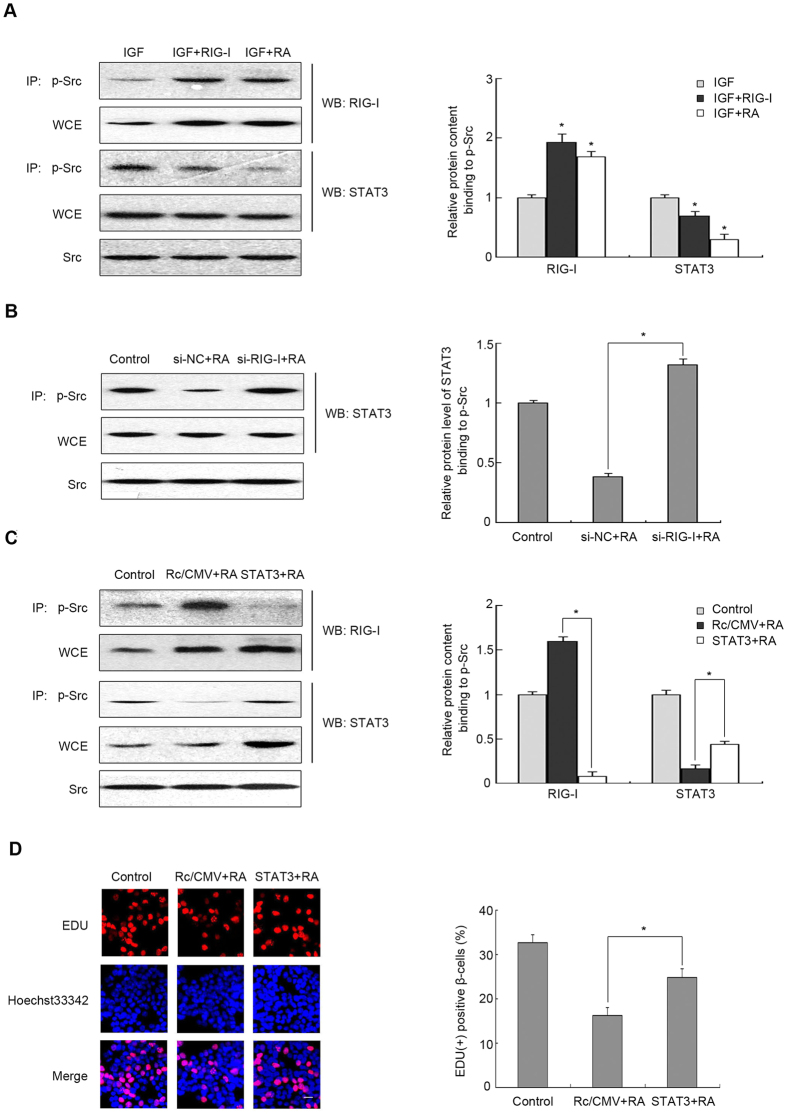
RIG-I interrupts the binding between Src and STAT3. (**A**) 24 h after transfection of a plasmid encoding *RIG-I*, Min6 cells were treated with IGF-1 or RA, as indicated. (**B**) RA was used to treat Min6 cells after 24 h transfection of si-RIG-I. (**C**) Min6 cells were transfected with STAT3 for 24 h, and then treated with RA. All these prepared extracts were immunoprecipitated with an antibody against p-Src. Immunocomplexes or whole cell extracts (WCE) were analysed by western blotting with antibodies against RIG-I or STAT3, as indicated. (**D**) Min6 cells were treated as in panel (**C)**, and DNA synthesis was analysed using EdU labelling assays. Representative micrographs of EdU labelling assays in MIN6 cells are shown (scale bar = 120 μm). The percentage of EdU-positive β cells was quantified. Data are means ± SEM of three separate experiments. *P < 0.05 versus control.

STAT3 is the downstream substrate of Src and binds to its SH2 domain corresponding sites. Src induces STAT3 dimerization and entry into the nucleus, resulting in the activation of the transcription and translation of target genes. To explore role of RIG-I in its competitive binding with Src and inhibition of Src activity^34^, MIN6 cells were grown in serum-free DMEM and treated with IGF. Western blotting was performed to detect binding of RIG-I and STAT3 with activated Src after co-immunoprecipitation with specific p-Src antibodies. After RIG-I transfection or RA treatment, binding of active Src with RIG-I increased, while binding of active Src with STAT3 was attenuated (Fig. 9A, P < 0.05). To investigate whether RIG-I modulated the Src and STAT3 interaction, the si-RIG-I plasmid was used in addition to RA treatment. Binding of active Src with STAT3 was increased compared to RA use only (Fig. 9B, P < 0.05). Furthermore, the STAT3 plasmid was also used to confirm these observations. Binding of active Src with RIG-I was inhibited and binding of active Src with STAT3 was not decreased after transfection with the STAT3 plasmid and treatment with RA compared with treatment with RA only (Fig. 9C, P < 0.05). Interestingly, the total Src level was not altered in these three experiments. EdU experiments were also carried out. Transfection with plasmid STAT3 could reverse the decrease in the percentage of EdU-positive β cells induced by RA (Fig. 9D, P < 0.05). These results suggested that RIG-I inhibits the Src/STAT3 association via competitive binding with active Src, leading to inhibition of pancreatic β cells proliferation.

## Discussion

As a metabolic disease, T2DM is characterized by insulin secreted from pancreatic β cells that cannot meet the demands of insulin sensitivity or insulin resistance, which might result from over-nutrition with chronic hyperglycaemia and hyperlipidaemia. In the state of excess nutrition, proliferative modulator Src is activated in diabetic islets^35^,^36^, which was confirmed by our data (Fig. 1). However, such a state did not promote β-cell proliferation, and did not increase the compensatory function (Fig. 2). Perhaps in the initial stage of over-nutrition, activated Src exerts a positive effect on β cell growth; however, with development of glucolipotoxicity and metaflammation, Src signalling is impaired by other negative modulators.

The two systems of metabolism and immunity are so integrated that more and more pattern recognition receptors are being identified to play important roles in metabolic disorders^37^. For example, activated PKR and elevated TLR3 upon glucolipotoxicity and pro-inflammatory cytokines stimuli both inhibit β cell proliferation to downregulate their functions, leading to failure of β cell compensation in the progression of type 2 diabetes^23^,^28^,^38^. RIG-I is another PRR thatthat is present in islet β cells. As shown in Fig. 3, RIG-I was significantly upregulated in the models of T2DM. Previous studies indicated that TNF-α, a cytokine that regulates innate immune responses, induced the expression of RIG-I in endothelial cells. The ability of TNF-α to upregulate RIG-I required protein synthesis, expression of functional type I IFNRs, and STAT1 signalling^39^. Hyperglycaemia causes oxidative stress in pancreatic beta cells of T2DM^40^. ROS might enhance RIG-I levels in pancreatic beta cells^41^. Thus, it necessary to determine whether Rig-I is a negative regulator involved in Src signals and β-cell proliferation.

Before exploring the potential association between RIG-I and Src-induced proliferation in pancreatic β cells, we obtained insights into the effect of RIG-I on β cell growth. Previous reports suggested that RIG-I mediated β cell lesions^42^; we showed that elevated RIG-I decreased β cell viability and abrogated IGF-stimulated proliferation through cycle arrest at the G1 phase. By contrast, knockdown of RIG-I could alleviate RA-mediated proliferation inhibition (Fig. 4). The results revealed that RIG-I is involved in inhibition of β-cells proliferation elicited by IGF-Src signals.

Recent studies have established that RIG-I restrains myeloid progenitor proliferation through competitive binding with active Src^4^. The recognition between RIG-I and Src acts through a cooperative interaction involving RIG-I recruitment domains (CARDs) and the Src SH1 domain. In inactive Src, the SH1 domain faces inward and is inaccessible to outside factors; therefore, the RIG-I CARDs preferentially associates with activated Src with an exposed SH1 domain. C-terminal to the RIG-I CARDs there is a classic PxxP motif. The PxxP motif can combine with the Src SH3 domain to prevent activated Src from inducing proliferation. Consistent with this finding,our results suggested that RIG-I could bind active Src competitively to inhibit pancreatic β cells proliferation elicited by IGF-Src signals (Figs 4 and 8).

RIG-I levels seemed to correlate with augmented β cells at G1 phase and reduced levels at S phase in a pancreatic β-cell line (Fig. 2), which was associated with several cell cycle modulators, such as cyclin D, P21, P27 and P53. In our experiments, accumulated RIG-I increased the protein level of P27, but there was no change at the mRNA level, suggesting post-translational regulation. Additionally, we found that MG132 could reverse significantly the decline of P27 in β cells without RIG-I function (Fig. 5). This evidence indicated that RIG-I-induced accumulation of P27 could be attributed to impaired ubiquitin-dependent proteasome degradation of P27. A similar mechanism underlying the degradation of P27 has been reported previously. For example, Prasad *et al*. demonstrated cytoplasmic P27 accumulation after treatment with MG132 in primary cervical cancer samples and cervical cancer-derived cell lines^43^.

Mounting evidence suggests that the S-phase kinase-associated protein 1 (SKP1)/CUL1/F-box protein Skp2 acts as an E3 ubiquitin ligase and is involved in the ubiquitination and proteasome-dependent degradation of P27^44^,^45^,^46^,^47^. Our analyses suggested the participation of Skp2 in RIG-I-mediated P27 stability. Correspondingly, Skp2 was downregulated by elevated RIG-I and glucolipotoxicity-stimulated P27 stability was abrogated when Skp2 was overexpressed (Fig. 6). Skp2 has been suggested to play a critical and specific role in regulating the cellular abundance of P27, and acts as an essential determinant of β cell proliferation. For example, diminished β cell mass, hypoinsulinaemia and glucose intolerance occurred in Skp2(−/−) mice^48^.

Meanwhile, several studies indicated that the transcript level of *SKP2* depends on STAT3. STAT3 is the most well characterized signalling pathway for increasing the nuclear localization and transactivation of genes, and is thought to promote cell growth and survival through multiple mechanisms, including increased expression of oncogenes, such as c-myc, Skp2 and cyclin D1. STAT3 can regulate the transcription of Skp2 by recruiting p300 and then binding to the enhancer region of the *SKP2* gene. Our studies indicated that STAT3 controlled *SKP2* gene expression as an important regulator in pancreatic β-cells, in which STAT3 overexpression can reverse the decrease in the Skp2 protein levels elicited by RIG-I (Fig. 7).

STAT3 is also the downstream substrate of Src^49^,^50^,^18^. STAT3 can be activated by tyrosine phosphorylated Src and is then translocated to the nucleus. In the nucleus, STAT3 dimmer and tetramers bind to specific DNA response elements to induce or repress gene transcription. The transcriptional activity of STAT3 was suppressed in *in vivo* models of T2DM^51^,^52^. Therefore, we hypothesized that elevated RIG-I might interrupt the binding of activated Src and STAT3. The hypothesis was confirmed by the co-immunoprecipitation and immunofluorescence assays, which showed that the physical interaction between RIG-I and phospho-Src resulted in little or no binding between STAT3 and phospho-Src and that elevated RIG-I inhibited the phosphorylation of STAT3 and its translocation to the nucleus (Figs 8 and 9). Furthermore, overexpression of STAT3 could compete with RIG-I to bind with activated Src and reverse the glucolipotoxicity-induced decrease of Skp2, thereby increasing the level of P27 to abrogate the RIG-I-dependent antiproliferative effect (Figs 7 and 9). The data showed that RIG-I competitively binds active Src and then inhibits the interaction between Src and STAT3, abrogating their proliferation effect in models of T2DM.

In summary, RIG-I is significantly activated by glucolipotoxicity and metaflammation, and is implicated in β cell mass loss through proliferation inhibition, leading to body decompensation in the course of T2DM. Via its PxxP motif, elevated RIG-I competes with STAT3 for activated Src and then blocks the Src/STAT3/Skp2 pathway to accumulate P27 function in pancreatic β cells, leading to cell cycle arrest at the G1 phase. These data provide a new insight into the pathogenesis of T2DM and suggest RIG-I as a novel drug target.

## Additional Information

**How to cite this article**: Pan, Y. *et al*. RIG-I inhibits pancreatic β cell proliferation through competitive binding of activated Src. *Sci. Rep.* **6**, 28914; doi: 10.1038/srep28914 (2016).

## Supplementary Material

Supplementary Information
