# Supplementary material for: RIG-I inhibits pancreatic β cell proliferation through competitive binding of activated Src
Source: Sci Rep. 2016 Jun 28;6:28914. doi: 10.1038/srep28914 (PMC4923948; doi:10.1038/srep28914)
Supplement: Supplementary Information [file srep28914-s1.pdf]

RIG-I inhibits pancreatic β cell proliferation through competitive binding of activated Src

Yi Pan[1]*, GuangMing Li[1,2]*, HengGao Zhong[1,3], MeiJuan Chen[4], TingTing Chen[1], LiLi Gao[1], HuiWen Wu[5], Jun Guo[1#]

1 Department of Biochemistry and Molecular Biology, Nanjing Medical University, Nanjing, PR China

2 Department of Anesthesiology, Huaian First People's Hospital, Nanjing Medical University, Huaian, Jiangsu, PR China

3 Medical Center for Digestive Diseases, Second Affiliated Hospital, Nanjing Medical University, Nanjing, Jiangsu, PR China

4 The Pre-clinical Medicine College, Nanjing University of Chinese Medicine, Nanjing, PR China

5 Laboratory Center for Basic Medical Sciences, Nanjing Medical University, Nanjing, PR China

* The authors contributed equally to this paper

[#] Corresponding author: Guoj@njmu.edu.cn; Guoj69@aliyun.com

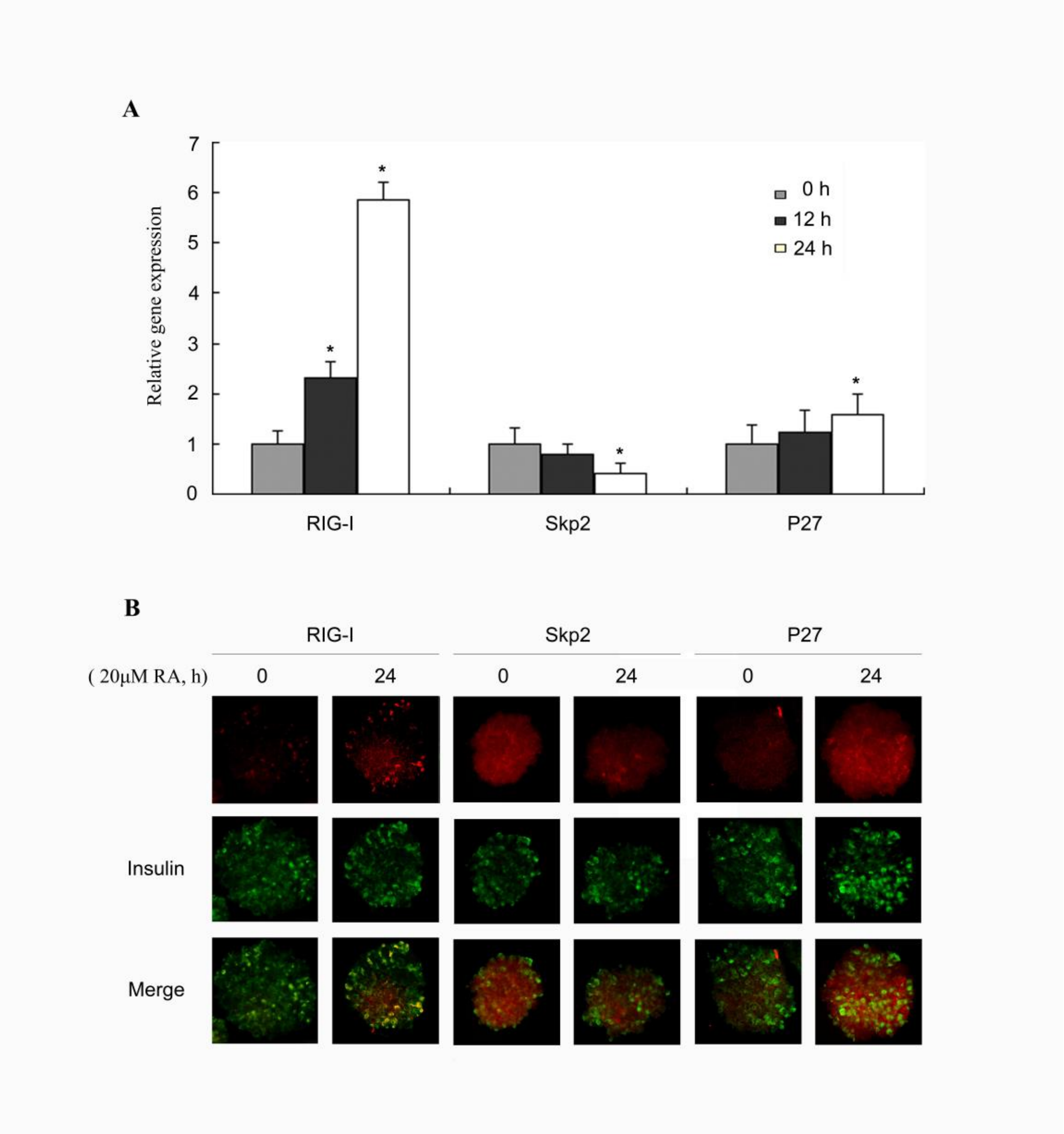

Figure S1: Expression of RIG-I, Skp2, and p27 in retinoic acid stimulated primary islet cells. Primary islet cells were treated with retinoic acid (RA) for the indicated times. (A) QRT-PCR was performed to detect the mRNA levels of *RIG-I*, *SKP2*, and *P27* in primary islet cells. (B) IFA was performed with antibodies directed against RIG-I, Skp2, or p27 (red) and insulin (green). Insulin with green staining was used to identify β cells (scale bar = 100μm). Data are means ± SEM of three separate experiments. *P < 0.05 versus control

Table S1. Real-time qRT-PCR primers sequences

Gene	Primer	Primer Length (bp)
cyclin D1 (mouse)	F 5'-GCGTACCCTGACACCAATCT-3'	20
	R 5'-CACAACTTCTCGGCAGTCAA-3'	20
cyclin E (mouse)	F 5'-GCTTCTAGACCTGTGCGTCC-3'	20
	R 5'-CTTTCTTTGCTTGGGCTTTG-3'	20
CDK2 (mouse)	F 5'-TTTGCTGAAATGGTGACCCGC-3'	21
	R 5'-ATCTTCATCCAGGGGAGGCA-3'	20
P27(mice)	F 5'-GATACGAGTGGCAGGAGGTG-3'	20
	R 5'-TTCTGTTCTGTTGGCCCTTT-3'	20
P21(mice)	F 5'-TCCAGACATTCAGAGCCACA-3'	20
	R 5'-GACCCAGGGCTCAGGTAGA-3'	19
Skp2 (mouse)	F 5'-ATGGACTGCTCTCAAACCTCG-3'	21
	R 5'-CCTGGAAAGTTCTCCCGACTAA-3'	22
RIG-I (mouse)	F 5'-GGACGTGGCAAAACAAATCAG-3'	21
	R 5'-GCAATGTCAATGCCTTCATCA-3'	21
β-Actin	F 5'-TCTACAATGAGCTGCGTGTG-3'	20
	R 5'-GGGGTGTTGAAGGTCTCAAA-3'	20
